# Quercetin Induces Mitochondrial Biogenesis through Activation of HO-1 in HepG2 Cells

**DOI:** 10.1155/2013/154279

**Published:** 2013-10-31

**Authors:** Nabin Rayamajhi, Seul-Ki Kim, Hiroe Go, Yeonsoo Joe, Zak Callaway, Jae-Gu Kang, Stefan W. Ryter, Hun Taeg Chung

**Affiliations:** ^1^School of Biological Sciences, University of Ulsan, Ulsan 680-749, Republic of Korea; ^2^Chitwan Medical College, Bharatpur-10, Chitwan, Nepal; ^3^Korea Bio Medical Science Institute, Seoul 135-818, Republic of Korea; ^4^Graduate School of Oriental Medicine, University of Wonkwang, Iksan 570-300, Republic of Korea; ^5^Department of Pulmonary and Critical Care Medicine, Brigham and Women's Hospital, Harvard Medical School, Boston, MA 02115, USA

## Abstract

The regeneration of mitochondria by regulated biogenesis plays an important homeostatic role in cells and tissues and furthermore may provide an adaptive mechanism in certain diseases such as sepsis. The heme oxygenase (HO-1)/carbon monoxide (CO) system is an inducible cytoprotective mechanism in mammalian cells. Natural antioxidants can provide therapeutic benefit, in part, by inducing the HO-1/CO system. This study focused on the mechanism by which the natural antioxidant quercetin can induce mitochondrial biogenesis in HepG2 cells. We found that quercetin treatment induced expression of mitochondrial biogenesis activators (PGC-1**α**, NRF-1, TFAM), mitochondrial DNA (mtDNA), and proteins (COX IV) in HepG2 cells. The HO inhibitor SnPP and the CO scavenger hemoglobin reversed the effects of quercetin on mitochondrial biogenesis in HepG2 cells. The stimulatory effects of quercetin on mitochondrial biogenesis could be recapitulated *in vivo* in liver tissue and antagonized by SnPP. Finally, quercetin conferred an anti-inflammatory effect in the liver of mice treated with LPS and prevented impairment of mitochondrial biogenesis by LPS *in vivo*. These salutary effects of quercetin *in vivo* were also antagonized by SnPP. Thus, our results suggest that quercetin enhances mitochondrial biogenesis mainly via the HO-1/CO system *in vitro* and *in vivo*. The beneficial effects of quercetin may provide a therapeutic basis in inflammatory diseases and sepsis.

## 1. Introduction

Mitochondrial biogenesis plays an important role in cell survival and repair [1–3]. Increased oxidative damage and inflammation can cause mitochondrial damage that may lead to serious acute and chronic pathologies such as multiorgan failure, neurodegeneration, and cardiovascular disease [3–6]. Mitochondrial biogenesis can enhance cellular function and survival *in vivo* and *in vitro* and promote cellular recovery from damage caused by adverse environmental, pathophysiological, and/or infectious agents [7, 8].

Mitochondrial biogenesis is regulated by a complex network of factors. The peroxisome proliferator-activated receptor gamma coactivator (PGC) family of transcription co-activators (e.g., PGC-1*α*) coactivate nuclear respiratory factor 2 (NRF-2/GA-Binding protein-A) and nuclear respiratory factor-1 (NRF-1) [1, 9]. PGC-1*α* and NRF-1 activate mitochondrial transcription factor A (TFAM) that is responsible for transcribing nuclear encoded mitochondrial proteins, including structural proteins as well as proteins involved in mitochondrial DNA (mtDNA) transcription, translation, and repair [1, 2, 8–11].

Quercetin is a naturally occurring flavonoid which has a broad spectrum of bioactive effects. Among these, quercetin can impact mitochondrial biogenesis by modulating enzymes and transcription factors in the inflammatory signaling cascade [10, 12]. Previous studies have shown that quercetin can increase messenger RNA (mRNA) for PGC-1*α*, the cytosolic deacetylase SirtI, and cytochrome *c* concentration in soleus muscles [13]. Quercetin, a potent phenolic antioxidant, can also modulate mitochondrial biogenesis by reducing ROS production in various cell types [14, 15]. Mitochondrial ROS can perturb cellular oxidant/antioxidant balance and participate in redox signaling. Oxidative stress-related ROS production can stimulate adaptive responses, such as Nrf2 translocation and binding to antioxidant response element (AREs) motifs in protective phase II antioxidant genes including heme oxygenase-1 (*Hmox1*). However, increased ROS production can cause mitochondrial dysfunction and cell death [9, 16]. Polyphenol antioxidants can prevent ROS-induced cellular damage by scavenging free radicals. The process of excess ROS elimination and mitochondrial biogenesis is connected with innate cellular antioxidant defense mechanisms. 

Heme oxygenase-1 (HO-1) is an important antioxidant enzyme that catalyses the rate-limiting step in heme-degradation. HO-1 induction protects against prooxidant heme release induced by many agents like LPS, cytokines, and ROS. Degradation of heme results in production of biliverdin, iron, and CO which have important physiological effects. Biliverdin is converted to the potent endogenous antioxidant bilirubin by NADPH: biliverdin reductase. Humans with HO-1 deficiency exhibit severe medical conditions such as anemia, leukocytosis, and hyperlipidemia, while animal models with HO-1 deficiency are susceptible to endotoxemia and chronic hypoxia [17, 18]. HO-1 deficient endothelial cells display increased injury in the presence of oxidative challenge, suggesting that the HO-1 pathway is a key cytoprotective mechanism against oxidative stress which contributes to cellular homeostasis [11, 17–19].

Endogenous CO contributes to the protective effects of HO-1 by modulation of the inflammatory response. CO binds to cytochrome *c* oxidase resulting in increased mitochondrial ROS production, which enhances mitochondrial biogenesis. Limited bioavailability of CO by hemoglobin treatment triggers cell death with a concomitant decline in ATP production, and mitochondrial generation of ATP significantly declined when CO availability was limited. These results suggest that CO, an enzymatic byproduct of HO-1 activity, is responsible for the function of HO-1 and that the HO-1/CO system may preserve mitochondrial biogenesis [17–20].

 In the current study we demonstrate the role of the HO-1/CO system in mediating mitochondrial biogenesis induced by the antioxidant quercetin in HepG2 cells. An understanding of the mechanisms underlying mitochondrial biogenesis may facilitate the development of therapeutics in diseases involving mitochondrial dysfunction (e.g., sepsis, and metabolic syndrome).

## 2. Materials and Methods

### 2.1. Reagents

Quercetin, Hemoglobin (Hb), and bacterial lipopolysaccharide (LPS, from *Escherichia coli* 055:B5) were purchased from Sigma-Aldrich (St Louis, MO). Tin protoporphyrin-IX (SnPP) was from Porphyrin Products Inc. (Logan, UT). Antibodies against *β*-actin were purchased from Santa Cruz Biotechnology (Santa Cruz, CA), and antibodies to cytochrome oxidase subunit IV (COX) IV and *α*-tubulin were purchased from Cell Signaling (Danvers, MA). Antibody against HO-1 was purchased from Assay Designs (Ann Arbor, MI). All other chemicals were purchased from Sigma-Aldrich. 

### 2.2. Cell Culture and Quercetin Treatment

HepG2 cells were purchased from ATCC (Manassas, VA). Cells were cultured in DMEM media supplemented with 10% fetal bovine serum, 100 U/mL penicillin, and 100 mg/mL streptomycin (Gibco, NY). Cells were maintained in a humidified incubator at 37°C under an atmosphere of 5% CO_2_. For quercetin treatment, HepG2 cells (4 × 10^4^ cells/well) were grown on 6-well plates overnight and quercetin was administered at various doses (5–25 *μ*M) and times (3–24 hrs).

### 2.3. Animals

All experiments with mice were approved by the Animal Care Committee of the University of Ulsan. Seven week-old male C57BL/6 mice were purchased from ORIENT (Pusan, Korea). The mice were maintained under specific pathogen-free conditions at 18–24°C and 40–70% humidity, with a 12 h light-dark cycle, and food and drinking water were available *ad libitum*.

C57BL/6 mice were treated with an intraperitoneal (*i.p.*) injection of quercetin (50 mg/kg) dissolved in 0.5% DMSO/PBS solution for seven alternate days. The control group of mice received the same amount of 0.5% DMSO/PBS solution. In some experiments, SnPP (50 *μ*mol/kg) was administered intraperitoneally (*i.p.*) to mice before quercetin injection. SnPP was dissolved in 0.1 N NaOH and diluted with PBS (pH 7.4). To study sepsis in mice, twenty-four hours after the final injection of quercetin, mice received an injection of LPS (10 mg/kg, *i.p.*). At 24 h after LPS injection, mice were sacrificed under anesthesia and liver tissue was harvested for RNA, mtDNA, and protein measurements.

### 2.4. Reverse Transcription Polymerase Chain Reaction (RT-PCR)

Total RNA of HepG2 was extracted using Trizol reagent (Invitrogen, CA). Two micrograms of total RNA were used for reverse transcription polymerase chain reaction (RT-PCR) analysis using oligo-dT primers (Qiagen, CA) and M-MLV reverse transcriptase (Promega, WI) according to the manufacturer's instructions. The forward and reverse primers used in the present study are shown in Table 1. PCR products were electrophoresed on 1.5% agarose gel and visualized by ethidium bromide staining. GAPDH cDNA level was used as an internal control.

### 2.5. Western Blotting Analysis

Cells were harvested in lysis buffer [25 mMTris-HCl (pH 7.5), 137 mM NaCl, 2.7 mM KCl, 1% Triton X-100] containing protease and phosphatase inhibitors cocktail (Sigma-Aldrich, St. Louis, MO). Protein concentration was measured with BCA protein assay reagent (Pierce, Rockford, IL). Equal amounts of proteins were separated using SDS-PAGE and transferred to polyvinylidene difluoride membranes (Thermo Scientific, Rockford, IL). Membranes were blocked with 5% skim milk in PBS containing 0.1% Tween 20 (PBS-T) for 1 h and then incubated with the specified antibodies. Signals were detected using the ECL detection system (Thermo Scientific, Rockford, IL, USA).

### 2.6. Quantitative Real-Time PCR Analysis of mt DNA Content

Genomic DNA (containing both mitochondrial and nuclear DNA) was isolated from cells using a Blood and Cell Culture DNA Mini Kit (Qiagen, Valencia, CA) according to the manufacturer's instructions. mtDNA was determined by SYBR green quantitative PCR (qPCR). The following primers for mtDNA were used: Human Complex II (succinate-ubiquinone oxidoreductase): forward primer 5′-CAAACCTACGCCAAAATCCA-3′ reverse primer 5′-GAAATGAATGAGCCTACAGA-3′. Mouse cytochrome *b* (*Mus musculus domesticus* mitochondrion): forward primer 5′-CCACTTCATCTTACCATTTA-3′ reverse primer 5′-ATCTGCATCTGAGTTTAATC-3′. The following primers for nuclear DNA were used: human *β*-actin: forward primer 5′-TCACCCACACTGTGCCCATCTACGA-3′ reverse primer 5′-CAGCGGAACCGCTCATTGCCAATGG-3′ and mouse 18 S rRNA: forward primer 5′-GGGAGCCTGAGAAACGGC-3′ reverse primer 5′-GGGTCGGGAGTGGGTAATTT-3′. Reactions were performed with SYBR Green qPCR Master Mix (2X; USB production, Affymetrix) on an ABI 7500 Fast Real-Time PCR System (Applied Biosystems, Carlsbad, CA).

### 2.7. Statistical Analysis

Multiple mean values were compared using analysis of variance (ANOVA) with GraphPad Prism. Values presented are mean ± SD. ANOVA using *t*-tests was applied to compare the mean of each group with that of the control group. A *P* < 0.05 was considered to be statistically significant.

## 3. Results

### 3.1. Quercetin Induces the Expression of Activators and Mitochondrial Proteins Associated with Mitochondrial Biogenesis

**Figure 1:**
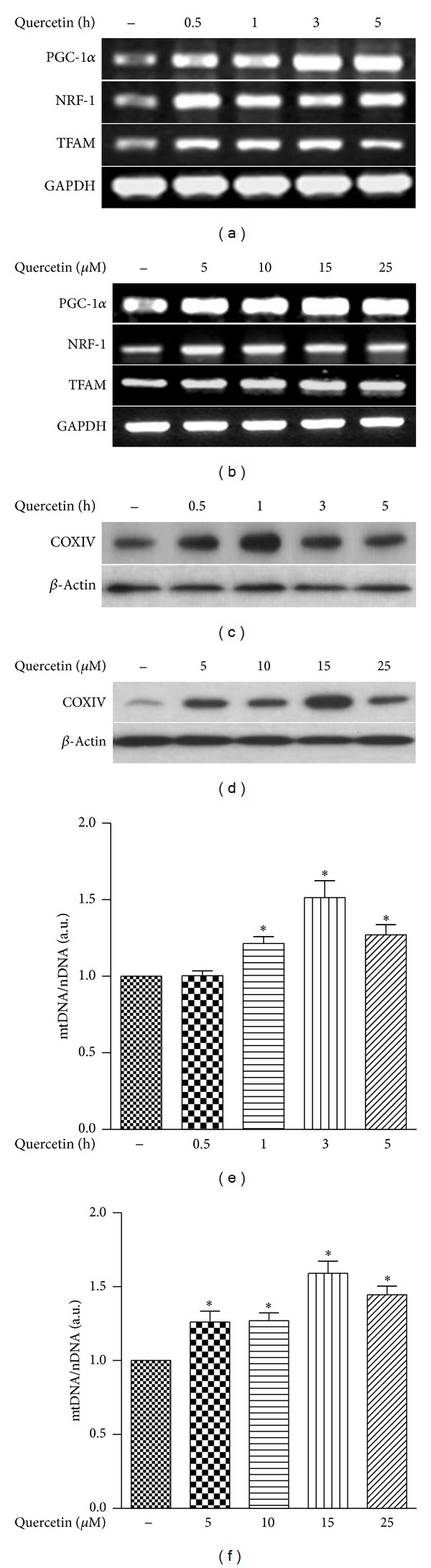
Time- and dose-dependent increases of mitochondrial biogenesis in HepG2 cells by quercetin treatment. (a–f) HepG2 cells (4 × 10^5^ cells/well) were exposed for indicated times (0, 0.5, 1, 3, 5 h) to various concentrations (0, 5, 10, 15, 25 *μ*M) of quercetin. (a, b) mRNA expressions of markers of mitochondrial biogenesis (PGC-1, NRF-1, and TFAM) were determined by reverse transcription PCR. GAPDH served as the standard. (c, d) Expression of COX IV protein was determined by Western blot analysis. *β*-actin served as the standard. (e, f) Expression of mitochondrial DNA (mtDNA) content was quantified by real-time PCR. Relative amount of mtDNA and nuclear DNA (nDNA) contents were compared. Results are expressed as mean ± SE of three independent experiments, and representative data are shown. *P < 0.05 compared with untreated control group. HepG2 cells (4 × 10^5^ cells/well) were exposed to quercetin 15 *μ*M, quercetin and SnPP (a), and Hb (b) for 3 h as described in panels (a) and (b). mRNA expressions of markers of mitochondrial biogenesis (PGC-1, NRF-1, and TFAM) were determined by reverse transcription (RT) PCR. Results are expressed as mean ± SE of three independent experiments, and representative data are shown. *P < 0.05 compared with untreated control group.

We determined the potential of quercetin to induce mitochondrial biogenesis by analyzing the mRNA expression levels of major regulators of mitochondrial biogenesis (i.e., PCG-1*α*, NRF-1, and TFAM). Treatment of HepG2 cells with quercetin (15 *μ*M) significantly increased the levels of PGC-1*α*, NRF-1, and TFAM mRNA in a time-dependent and dose-dependent manner (Figures 1(a) and 1(b)). Quercetin treatment also stimulated the expression of the major mitochondrial protein COX IV in a time- and dose-dependent manner (Figures 1(c) and 1(d)). Since increases of mtDNA copy number also represent an index of mitochondrial biogenesis, we measured the amount of mtDNA in HepG2 cells treated with quercetin (Figures 1(e) and 1(f)). Quercetin increased mtDNA copy number with an apparent maximum at a quercetin dose of 15 *μ*M for 3 hrs. 

### 3.2. Quercetin Induces Mitochondrial Biogenesis via Expression of the HO-1/CO System in HepG2 Cells

**Figure 2:**
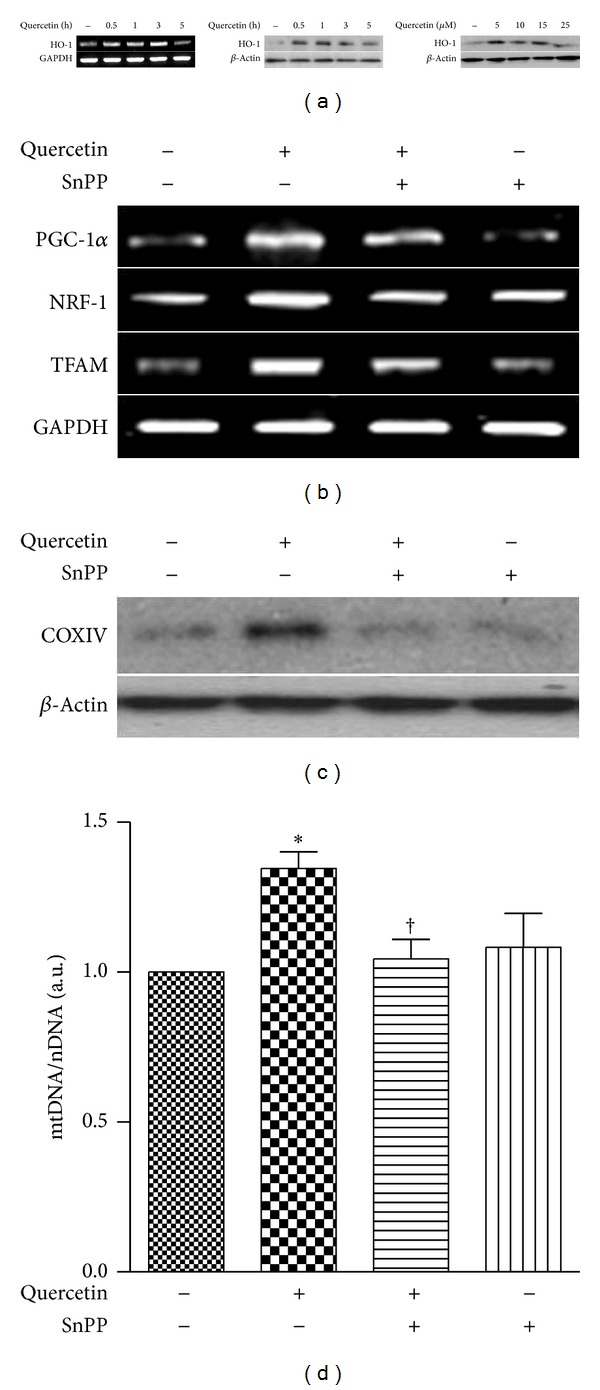
Induction of mitochondrial biogenesis by quercetin is regulated by activation of HO-1. (a) Expression levels of HO-1 mRNA and protein were determined after HepG2 cells were exposed for indicated times (0, 0.5, 1, 3, 5 h) and with the indicated concentrations (0, 5, 10, 15, 25 *μ*M) of quercetin. Expressions of HO-1 mRNA and protein were determined by RT-PCR and Western blotting. GAPDH and *β*-actin served as the standards, respectively. (b–d) HepG2 cells were exposed to 15 *μ*M of quercetin for 3 h with or without 20 *μ*M of SnPP. (b) Expressions of PGC-1, NRF-1, and TFAM mRNA were determined by RT-PCR. (c) Expression of COXIV protein was determined by Western blotting. (d) Expression of mtDNA content was quantified by real-time PCR. Relative amounts of mtDNA and nDNA contents were compared. Results are expressed as mean ± SE of three independent experiments, and representative data are shown. *P < 0.05 compared with untreated control group; ^†^ *P* < 0.05 compared with cells treated with quercetin alone.

Quercetin has previously been shown to induce HO-1 expression in various cell types, which may account in part for the cytoprotective, antiapoptotic, antioxidant, and anti-inflammatory effects of this compound [19, 21–23]. In the current study, we examined whether quercetin can induce the expression of HO-1 at the RNA or protein level in HepG2 cells. As shown in Figure 2(a), an increase in HO-1 mRNA and protein was detected at various times and doses of quercetin. The maximal effect of quercetin on HO-1 mRNA and protein expression was observed after treatment with 15 *μ*M for 3 h (Figure 2(a), left and middle). When HepG2 cells were treated with different concentrations of quercetin (5–25 *μ*M) for 3 hrs, the maximum induction of HO-1 protein was detected at 15 *μ*M (Figure 2(a), right). Thus, the increases of HO-1 expression achieved with quercetin treatment were consistent with previous reports [24–26]. 

**Figure 3:**
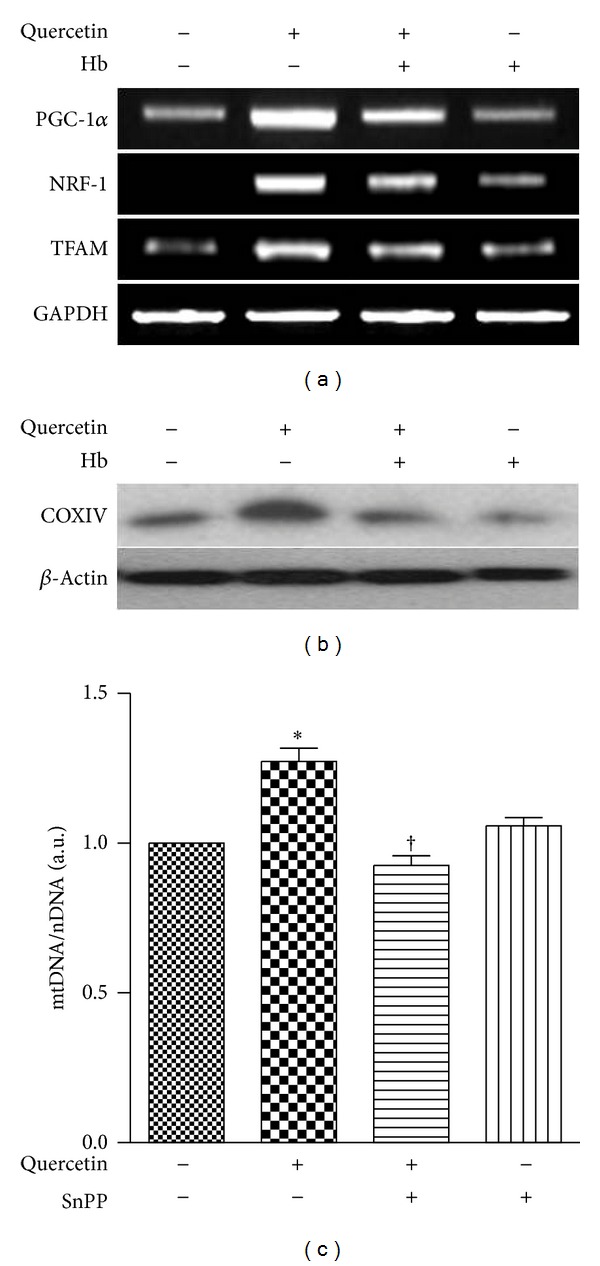
Quercetin induction of mitochondrial biogenesis requires CO. (a–c) HepG2 cells were exposed to 15 *μ*M of quercetin for 3 h with or without 20 *μ*g/mL of Hb. (a) Expression of PGC-1, NRF-1, and TFAM mRNA was determined by RT-PCR. (b) Expression of COX IV protein was determined by Western blotting. *β*-actin served as the standard. (c) Expression of mitochondrial DNA (mtDNA) content was quantified by real-time PCR. Relative amounts of mtDNA and nDNA contents were compared. Results are expressed as mean ± SE of three independent experiments, and representative data are shown. *P < 0.05 compared with untreated control group; ^†^ *P* < 0.05 compared with cells treated with quercetin alone.

It is also known that CO generated by HO-1 can activate mitochondrial biogenesis [27, 28]. Therefore, we hypothesized that the activation of mitochondrial biogenesis by quercetin also involves the activation of the HO-1/CO system in HepG2 cells. To investigate whether HO-1/CO is involved in quercetin-induced mitochondrial biogenesis, the competitive HO inhibitor tin-protoporphyrin-IX (SnPP) and hemoglobin (Hb), a CO scavenger, were employed with or without addition of quercetin, and expression levels of PGC-1*α*, NRF-1, and TFAM mRNA were evaluated. As shown in Figure 2(b), treatment of HepG2 cells with SnPP and quercetin resulted in reduced levels of PGC-1*α*, NRF-1, and TFAM mRNA expression levels compared with cells treated with quercetin alone. Quercetin-induced COX IV expression was also inhibited by SnPP treatment (Figure 2(c)). Likewise, the increase in mtDNA by quercetin was suppressed by SnPP treatment (Figure 2(d)). To evaluate the involvement of CO in quercetin-induced mitochondrial biogenesis, HepG2 cells incubated with quercetin were cotreated with Hb (Figure 3). The Hb treatment inhibited the increases of PGC-1*α*, NRF-1, and TFAM mRNA expression induced by quercetin in HepG2 cells (Figure 3(a)). Hb treatment decreased the expression of COX IV protein induced by quercetin (Figure 3(b)). Furthermore, the induction of mtDNA levels by quercetin was inhibited by treatment with Hb (Figure 3(c)).

**Figure 4:**
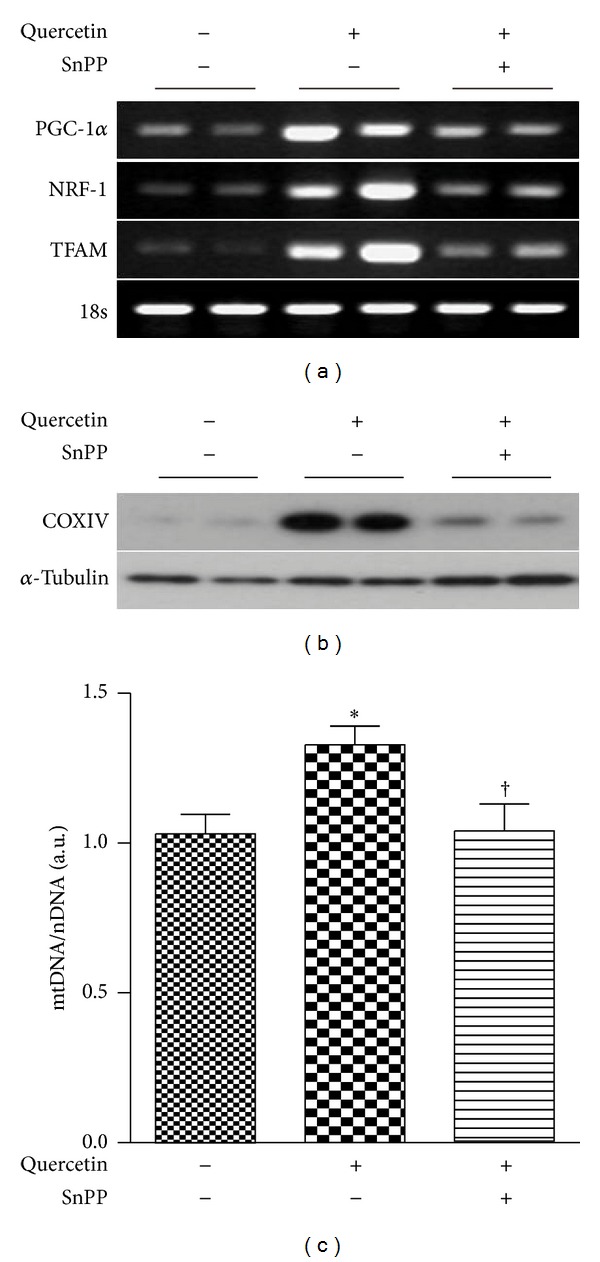
Quercetin induces mitochondrial biogenesis through the induction of HO-1/CO system *in vivo.* (a–c) C57BL/6 Mice were injected intraperitoneally (i.p.) with quercetin (50 mg/kg) for 7 alternate days, with or without SnPP (50 *μ*mol/kg) prior to injection with the addition of quercetin. Liver tissues were excised and analyzed for mitochondrial biogenesis in mice. Experimental analyses were performed with liver tissue. (a) Expressions of PGC-1, NRF-1, and TFAM in mRNA were determined by RT-PCR. 18 S rRNA served as the standard. (b) Expressions of COX IV protein were determined by Western blotting. *α*-tubulin served as the standard. (c) Expression of mtDNA content was quantified by real time PCR. Relative amounts of mtDNA and nDNA contents were compared. Results are expressed as mean ± SE of three independent experiments (*n* = 5/group), and representative data are shown. *P < 0.05 compared with the uninjected control group; ^†^ *P* < 0.05 compared with LPS injected mice group.

To examine the role of HO-1 in quercetin-induced mitochondrial biogenesis *in vivo*, quercetin was injected intraperitoneally in C57BL/6 mice for 7 alternate days. Mice were treated with SnPP prior to quercetin injection. In accordance with the results observed in HepG2 cells, quercetin increased the expression of PGC-1*α*, NRF-1, and TFAM mRNA, COX IV expression, and mtDNA *in vivo* (Figure 4). The cotreatment with SnPP inhibited quercetin-induced PGC-1*α*, NRF-1, and TFAM mRNA, COX IV expression, and mtDNA (Figure 4). Thus, these results suggest that HO-1/CO system is required for quercetin-induced mitochondrial biogenesis *in vitro* and *in vivo.*

### 3.3. Quercetin Restores LPS-Damaged Mitochondrial Integrity via HO-1/CO Induction

**Figure 5:**
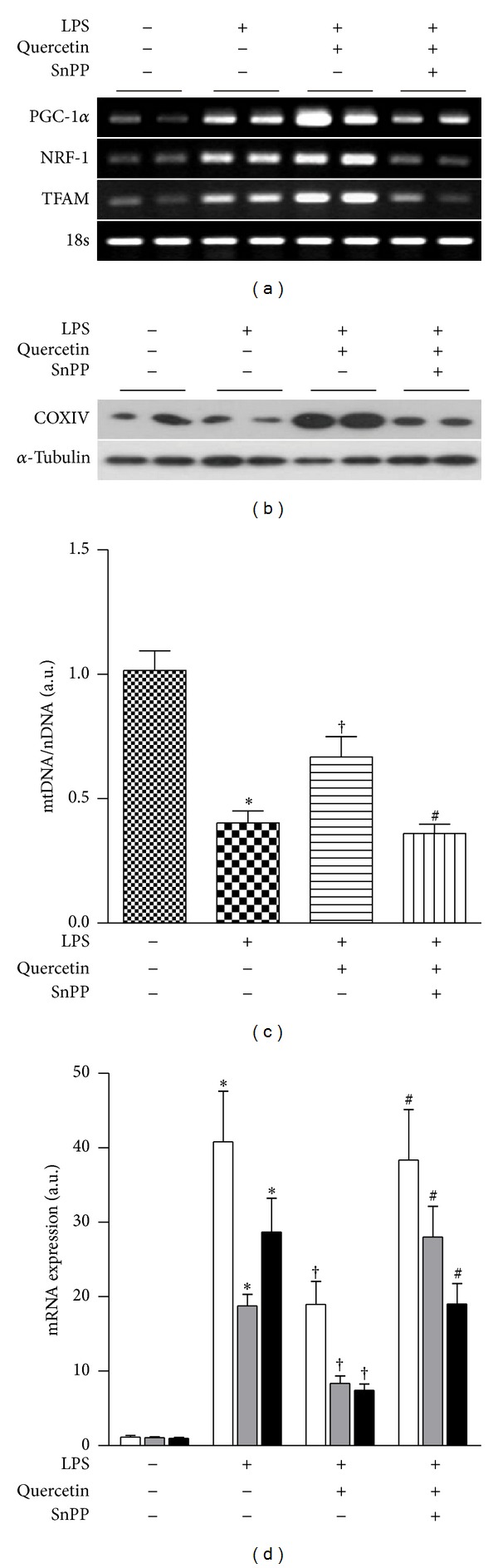
Quercetin restores mitochondrial biogenesis in LPS-treated mice in a HO-dependent fashion. (a to d) C57BL/6 mice were injected with quercetin (50 mg/kg) for 7 alternate days, with or without SnPP (50 *μ*mol/kg) prior to injection with quercetin, and then challenged for 24 hours with *i.p.* injection of LPS (10 mg/kg). (a) Expressions of PGC-1, NRF-1, and TFAM in mRNA were determined by RT-PCR. 18 S rRNA served as the standard. (b) Expression of COX IV protein was determined by Western blot analysis. *α*-tubulin served as the standard. (c) Expression of mtDNA content was quantified by real-time PCR. Relative amounts of mtDNA and nDNA contents were compared. (d) Expression of TNF*α*, IL-1*β*, and IL-6 mRNA was quantified by real-time PCR. 18 S rRNA served as the standard. Results are expressed as mean ± SE of three independent experiments (*n* = 5/group), and representative data are shown. *P < 0.05 compared with the uninjected control group; ^†^ *P* < 0.05 compared with LPS injected mice group; ^#^ *P* < 0.05 compared with mice in the LPS + quercetin group.

Finally, we examined whether quercetin could contribute to cellular protection against LPS-induced mitochondrial damage in a HO-1/CO-dependent manner. LPS treatment increased the expression PGC-1*α*, NRF-1, and TFAM mRNA in mouse liver. Moreover, quercetin treatment further increased the expression of PGC-1*α*, NRF-1, and TFAM mRNA after LPS treatment (Figure 5(a)). However, LPS treatment clearly diminished hepatic COX IV and mtDNA content (Figures 5(b) and 5(c)). In contrast, quercetin protected against the loss of COX IV and mtDNA content in LPS-treated animals. SnPP antagonized the protective effects of quercetin with respect to hepatic PGC-1*α*, NRF-1, and TFAM mRNA expression, COX IV expression, and mtDNA content in this model (Figures 5(a), 5(b), and 5(c)). LPS caused increases in the hepatic expression of TNF*α*, IL-1*β*, and IL-6 mRNA. Quercetin administration inhibited the LPS-dependent induction of TNF*α*, IL-1*β*, and IL-6. This effect of quercetin was in turn inhibited by SnPP injection (Figure 5(d)). These results suggest that quercetin restores mitochondrial integrity from LPS damage *via* activating the HO-1/CO system.

## 4. Discussion

Mitochondrial biogenesis has been the focus of extensive studies due to its beneficial effects in many health conditions related to performance, diabetes, neurodegeneration, the cardiovascular system, cancer, and infection. Death resulting from multiple organ failure (MOF) during severe sepsis and septic shock has been related to mitochondrial damage. Rescue of mice from lethal *Staphylococcus aureus *sepsis and protection against cardiomyocyte apoptosis have been linked to mitochondrial biogenesis induction [27, 28]. 

Quercetin is a polyphenolic compound that exerts several potent bioactivities including antiproliferative, anti-inflammatory, antioxidant, and immune system effects. Recent *in vitro* and *in vivo* experiments have shown that the salutary effects of quercetin may involve activation of mitochondrial biogenesis [11, 14, 15]. Previous research has shown positive effects of quercetin on endurance and health maintenance [4, 13, 29, 30]. These benefits may involve the antioxidant, anti-inflammatory, and psychostimulant effects of quercetin, as well as effects on mitochondrial biogenesis. Because abnormalities that contribute to impaired health or development of metabolic disorders are linked to mitochondrial dysfunction, the stimulation of mitochondrial biogenesis by quercetin may represent the most important bioactivity of this compound [1, 2, 10, 12, 31]. 

Our results demonstrate that quercetin can enhance the expression of PGC-1*α*, a master regulator of the transcriptional network that regulates mitochondrial biogenesis, in HepG2 cells. PGC-1*α* is responsible for activating the transcription of genes involved in oxidative phosphorylation and mtDNA replication. NRF-1 and NRF-2, which are transcription factors acting on nuclear genes coding for proteins necessary for the mitochondrial respiratory chain or for mtDNA transcription and replication, are also activated by PGC-1*α*. PGC-1*α* and NRFs coactivate the expression of TFAM, which is important for regulation and maintenance of mtDNA copy number [1, 2, 10]. Our results also showed increased expression of PGC-1-related transcription factors associated with mitochondrial biogenesis in HepG2 cells treated with quercetin (Figure 1). Similarly, the mitochondrial respiratory chain consisting of four membrane-bound complexes (Complex I–IV) involved in ATP synthesis and transfer of electrons formed by NADH or FADH_2_ is an indicator of mitochondrial biogenesis [32]. Increases in cytochrome *c*concentration typically occur in conjunction with similar increases in other mitochondrial enzymes of the electron transport chain and enzymes in the tricarboxylic acid cycle and *β*-oxidation pathway, that lead to an overall increase in mitochondrial capacity [13, 32]. Previous reports of quercetin-induced increases in mitochondrial biogenesis are consistent with the increases in COX IV protein expression observed in our study (Figures 1–3). The critical effects of quercetin on mitochondrial biogenesis *in vitro* have been demonstrated at a dose of 15 *μ*M quercetin. To study the effects of quercetin *in vivo*, animals were treated with a 50 mg/kg dose of quercetin. According to Ruiz et al. [33], treatment of mice with up to 3000 mg/kg quercetin did not cause any toxicity. Among the doses of quercetin (25, 50 and 100 mg/kg) tested in mice, we have found that 50 and 100 mg/kg doses of quercetin have a significant effect on mitochondrial biogenesis. However, one of the limitations of the current study is that the effect of oral administration of quercetin has not been tested. Further studies would be needed to determine the therapeutic benefit of oral quercetin in these systems.

Recent reports have also shown that the HO-1/CO system can stimulate mitochondrial biogenesis which may account in part for the cytoprotective effects of this system [27, 28]. Recent research has elucidated the role of HO-1 and CO in cellular defense mechanisms against oxidative damage. Quercetin has gained much attention because of its ability to confer cytoprotective effects through induction of HO-1 in various cell lines and primary hepatocytes [9, 11, 13, 17–20, 34]. CO, an enzymatic byproduct of HO-1, can mediate the cytoprotective effects of HO-1 activity. Our recent work has shown that endoplasmic reticulum (ER) stress caused significant decline of CO bioavailability that reduced mitochondrial ATP generation [9, 13, 17, 18, 35]. Similarly, in our current study, we have shown that the deleterious effect on mitochondria due to LPS administration was restored by quercetin. The beneficial effects of quercetin in the LPS model were in turn abrogated by SnPP. Thus, it is likely that quercetin induces mitochondrial biogenesis via the HO-1/CO system in HepG2 cell lines [18, 28, 35]. 

High glucose produces a high concentration of ROS that induces cellular dysfunction. Previous research in the human hematoma cell line, HepG2, has shown that hyperglycemia elicits detrimental changes in liver cells [5, 36]. Increased oxidative damage caused either by an overproduction of free radicals and ROS or by an impairment of the endogenous antioxidant defense system is well studied in epithelial cells and HepG2 cells [16, 19, 36]. Prevention of lung oxidative damage in acute lung injury/acute respiratory distress syndrome by quercetin has been shown to involve increases in HO-1 production [19]. In conclusion, we demonstrated that quercetin enhances cell survival against oxidative stress through an HO-1/CO-dependent increase in mitochondrial biogenesis. The antioxidant and mitochondrial biogenesis properties of quercetin may be helpful in developing therapeutic strategies to enhance cell survival during oxidative stress imposed by environmental and dietary factors. 

## Figures and Tables

**Table 1:** Gene primers used in this study.

Gene	Forward primer (5′-3′)	Reverse primer (5′-3′)
hPGC1*α*	GGAACTGCAGGCCTAACTCC	CACTGTCCCTCAGTTCACCG
hNRF-1	CCAGTGGCCACACAGAACTC	CTTCCTTTCCCTTCCACTGC
hTfam	ATGCTTATAGGGCGGAGTGG	TGGTTTCCTGTGCCTATCCA
hHO-1	GGAACTTTCAGAAGGGCCAG	GTCCTTGGTGTCATGGGTCA
hGAPDH	GGGGCTCTCCAGAACATCAT	TCAAGGGGTCTACATGGCAA
mPGC-1*α*	GGAACTGCAGGCCTAACTCC	TTGGAGCTGTTTTCTGGTGC
mNRF-1	CTCCAAACCCAACCCTGTCT	TGGTGGCCTGAGTTTGTGTT
mTfam	CAGCCAGGTCCAGCTCACTA	ATTAGGAGGGTCTCGCTCCA
mTNF-*α*	AGACCCTCACACTCAGATCATCTTC	TTGCTACGACGTGGGCTACA
mIL-1*β*	TCGCTCAGGGTCACAAGAAA	ATCAGAGGCAAGGAGGAAACAC
mIL-6	CCAGAGATACAAAGAAATGATGG	ACTCCAGAAGACCAGAGGAAAT
m18S	CAGTGAAACTGCGAATGGCT	TGCCTTCCTTGGATGTGGTA
